# The Hydrogen Storage Properties and Catalytic Mechanism of the AZ31-WS_2_ Nanotube/Pd Composite

**DOI:** 10.3390/nano15110802

**Published:** 2025-05-27

**Authors:** Song-Jeng Huang, Veeramanikandan Rajagopal, Sakthipriya Balu, Sivakumar Selvaraju, Murugan Subramani

**Affiliations:** Department of Mechanical Engineering, National Taiwan University of Science and Technology, Taipei City 10607, Taiwand10803802@mail.ntust.edu.tw (S.B.);

**Keywords:** hydrogen storage, magnesium alloys, inorganic nanotubes, transition metals, catalytic enhancement

## Abstract

Magnesium-based alloys, known for their high hydrogen storage capacity, suffer from sluggish kinetics and high activation energy barriers. It can be further optimized through synergistic combinations with metal hydrides. This study aims to address these limitations by investigating the hydrogen sorption properties of AZ31 magnesium alloy combined with different compositions of WS2 nanotubes (NTs) and Pd. The materials AZ31, WS2 (tungsten disulfide) NTs, and Pd were pre-processed via the mechanical ball milling process. Field emission-scanning electron microscopy (FE-SEM) and transmission electron microscopy (TEM) were employed to investigate the composite morphology and confirm the nanotubular structure of WS_2_. This work is among the first to explore the synergistic catalytic effects of WS_2_ nanotubes and Pd on the hydrogenation/dehydrogenation behavior of AZ31 alloys. The composite with 8 wt.% WS_2_ NT/Pd demonstrated the fastest hydrogen sorption kinetics and a significant reduction in activation energy, from 123.25 kJ/mol to 104.58 kJ/mol. These results highlight the enhanced dehydrogenation performance of AZ31 through catalyst inclusion, offering a promising approach to improve hydrogen storage materials. These findings highlight the potential of combining inorganic NTs and transition metals as effective catalysts to enhance the hydrogen storage performance. This research paves the way for developing advanced hydrogen storage materials with improved performance, contributing to a sustainable energy future.

## 1. Introduction

Hydrogen energy is regarded as one of the best prospects for creating a carbon–free civilization due to its environmental friendliness, high energy density, non-toxicity, and repeatability. However, the advancement of a green hydrogen society is still constrained by the lack of a safe, high-density storage technology. In terms of security and density, solid-state hydrogen storage is preferable to liquid hydrogen storage at cryogenic temperatures (−253 °C, 0.5–1 MPa) and gaseous hydrogen storage at high pressure (35–70 MPa at ambient temperature) [1,2,3,4,5,6]. Magnesium hydride (MgH_2_) is one of several solid-state hydrogen storage materials that has received great attention due to its large volumetric (110 g/L) and gravimetric (7.6 wt.%) capacity, abundance of the magnesium element, and low price. The use of MgH_2_ is unfortunately constrained by the sluggish rates of hydrogen absorption and desorption as well as the high desorption temperature [7,8,9,10,11,12]. The hydrogen absorption and desorption properties of MgH_2_ under moderate circumstances have recently been tuned using methods such as surface modification, nano-structuring, combining with other additives, and catalyst doping to overcome the aforementioned issues. Among them, adding a catalyst is one of the most practical modifying techniques, which has greatly contributed to the improved de/hydrogenation kinetics of MgH_2_ [13,14,15,16].

In recent years, the catalytic effect of transition metal dichalcogenides (TMDs) and transition metal sulfides on MgH_2_ has been reported previously. MgH_2_ catalyzed by WS_2_ was reported in 2015 and demonstrated an outstanding catalytic action on MgH_2_. The results showed that the W and MgS produced in the interaction between MgH_2_ and WS_2_ during ball milling acted as a catalyst to enhance the kinetic characteristics of MgH_2_ [17]. Jia et al. in 2013 [18], revealed that MgH_2_ added with 16.7 wt.% of MoS_2_ desorbed 0.57 wt.% hydrogen, whereas the ball-milled MgH_2_ only desorbed 0.1 wt.% of hydrogen within 10 min at 300 °C. They state that the Mo and MgS produced by the interaction of MgH_2_ and MoS_2_ might weaken the bonds between Mg-H and provide an active site for hydrogen diffusion. Additionally, NiS was added to MgH_2_ to provide a more favorable catalytic action. Moreover, they concluded that the catalytic impact of the produced MgS was still unidentified, with the exception of the catalytic action of Ni [19]. Zhang et al. (2018) [20] found that when FeS_2_ was introduced to MgH_2_, the hydrogen release increased from 0.18 wt.% to 1.24 wt.% in 1400 s at 300 °C. They proposed that the MgH_2_-FeS_2_ composite’s apparent activation energy might be greatly reduced by in situ-formed Fe, which could offer active sites and hydrogen diffusion channels. From the above reports, the de/hydrogenation kinetics and activation performance of MgH_2_ might be greatly improved by transition metals and sulfides. The performance of hydrogen absorption and desorption could be enhanced by in situ-formed metals like W and Fe. Particularly transitional metals such as W have a remarkable ability to weaken Mg-H bonds.

In addition, the hydrogen sorption kinetics were significantly accelerated by mixing different transition metal catalysts with magnesium and its alloys. Particularly, it is desirable to utilize transition metals (Ni, Fe, Cu, Ti, and V) as catalysts to accelerate the sorption of hydrogen atoms onto the surface of materials made of magnesium alloys. The addition of Ni in AZ31-Mg alloy improved the sorption properties of the alloy materials considerably. The rate of hydrogenation and dehydrogenation is increased due to the hydrogen molecules, which diffuse into the Mg crystal lattice quickly. Notably, even at a modest temperature, the presence of a catalyst improves the hydrogen storage ability [21,22,23,24,25,26,27,28,29,30]. When compared to other transition metals, the inclusion of Pd-related catalysts has shown a substantial catalytic effect on temperature-dependent hydrogen storage. The Pd nanoparticles included with multiwall carbon nanotubes involve three different sorption mechanisms in the temperature range of 110 K to 400 K [31].

In this work, we prepared AZ31-WS_2_ NT/Pd composite as hydrogen storage materials. WS_2_ NTs were synthesized through the chemical vapor deposition (CVD) method. The WS_2_ NT/Pd composite was prepared by adding the palladium (Pd) particles to the WS_2_ NT, which was further ball-milled with AZ31 magnesium alloy in order to improve the hydrogenation properties. The role of the synthesized WS_2_ NT/Pd composite on the activation process and the isothermal sorption properties was investigated, which could lead to the catalytic mechanism of transition metals with inorganic nanotubes in the future for magnesium-based hydrogen storage alloy materials. To the best of our knowledge, this is the first study to investigate the synergistic effects of WS_2_ NTs and Pd catalysts on the hydrogen storage properties of AZ31 magnesium alloy.

## 2. Experimental

### 2.1. Synthesis of WS_2_ Nanotubes

The tungsten disulfide (WS_2_) nanotubes (NTs) were synthesized in a solid–gas reaction process. The WO_3_ powder with a dimension of ~100 nm was used to produce the WS_2_ NTs. The WO_3_ powder was evenly spread on a quartz boat and kept in the reaction chamber. During the reaction, the temperature was maintained at 900 °C under a 1% H_2_/99% N_2_ gas environment for one hour with a flow rate of 400 mL min^−1^. Equation (1) illustrates the reduction of WO_3_ by H_2_ gas into the intermediate phase of WO_3−x_. After completing the reduction procedure, the reaction chamber was filled with 1% H_2_/99%N_2_ and H_2_S for 1 h to sulfurize the reduced WO_3−x_. The WS_2_ was formed as a result of the sulfurization process [32,33,34,35]. After cooling to ambient room temperature, WS_2_ NTs were collected from the reaction chamber. The WS_2_ formation from the WO_3−x_ phase was explained in Equation (2).

WO_3_ (s) + H_2_ (g) → WO_3−x_ + xH_2_O + (1 − x) H_2_ (1)

WO_(3−x)_ + (1 − x) H_2_ + H_2_S → WS_2_ + (3 − x) H_2_O. (2)

### 2.2. Synthesis of AZ31-WS_2_ NT/Pd Composite

The commercial magnesium-based AZ31 alloy (3.08 Al, 0.908 Zn, Mn 0.393 Bal Mg) was purchased from Kuangyue Co., Ltd., Taipei, Taiwan. The manually rasped alloy powders from the pure AZ31 magnesium alloy ingots were prepared by following the previous procedures [21,36]. Palladium (Pd) particles were purchased from Top Nano Technology Co., Ltd., Taipei, Taiwan with 99.9% purity.

The AZ31-WS_2_NT/Pd (AZ31-WP) composite particles with various weight ratios were mechanically milled by high-energy mechanical ball milling with the ball-to-powder ratio of 40:1, and the rotation speed was maintained at 400 rpm for 8 h of milling time. Three different types of compositions were prepared and denoted as AZ31-0 WP (0 wt.%), AZ31-4 WP (4 wt.%), and AZ31-8 WP (8 wt.%). The planetary-type ball-milling machine (Retsch PM 100, Haan, Germany) was used for the preparation of all the samples by the milling process. All the powder sample handling was carried out in an inert atmosphere.

### 2.3. Characterizations

The phase compositions and transformations of the prepared powder samples were examined by X-Ray diffraction (XRD, D2 phaser, Bruker, Billerica, MA, USA) with a Cu-Kα ray source fixed at a voltage of 45 kV and a current rating of 0.8 mA. The phase compositions were analysed by Diffract.EVA software V4.3. Microstructure and powder morphologies of AZ31-WS_2_ NT/Pd composite were observed by field emission electron microscopy (FE-SEM), model JSM 6390LV, equipped with an energy dispersive spectrometer (EDS). Morphologies of WS_2_ NTs were investigated by transmission electron microscopy (TEM), model Philips Technai F20 G2 FEI-TEM.

**Scheme 1:**
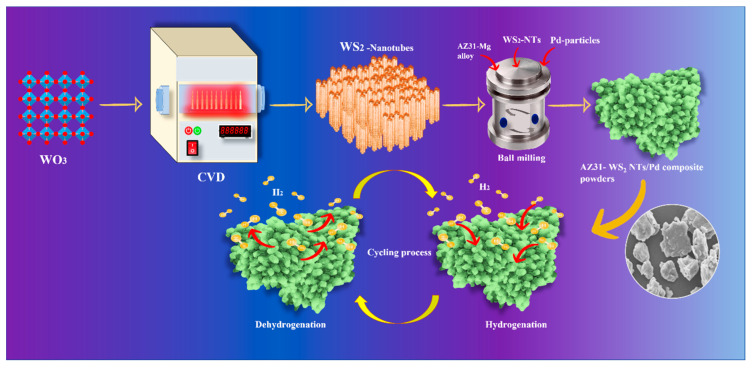
AZ31-WS_2_ NT/Pd composite materials synthesis and de/hydrogenation process.

Hydrogenation and dehydrogenation properties were measured using a Sievert-type apparatus, with operations managed by the LabView 2021 21.0 software program. A total of 0.2 g of ball-milled samples was located in the stainless-steel reactor. The activation process was conducted before the isothermal hydrogen testing. The isothermal hydrogenation testing was performed at a temperature of 375 °C. During the hydrogenation reaction, the initial hydrogenation pressures of 3 MPa for absorption and 0.0010 MPa for desorption were maintained. In order to obtain high-accuracy data, a high-precision pressure sensor and thermocouples were used to measure the pressure variation and the reaction temperature. Differential scanning calorimeter (DSC-MERRLER TOLEDO high temperature thermal analysis system) was used to conduct the sample decomposition analysis at different heating rates from 50 to 500 °C. The process of materials synthesis and de/hydrogenation process are displayed in Scheme 1.

## 3. Results and Discussion

### 3.1. Structural Analysis of WS_2_ NTs

**Figure 1:**
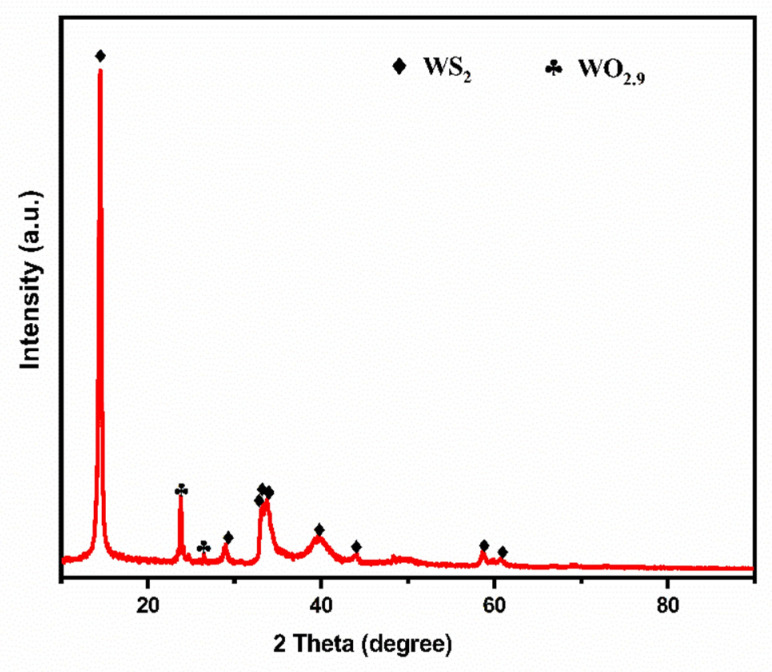
XRD pattern of as-synthesized WS_2_ NTs.

The XRD pattern of synthesized WS_2_ NTs is shown in Figure 1. The characteristic peaks at 14.6°, 33.9°, and 39.2° correspond to the planes of (002), (101), and (103) of WS_2_ NTs, respectively, demonstrating that the WS_2_ NTs were synthesized effectively using the chemical vapor deposition gas–solid reaction method. The sharp peaks indicate that the synthesized WS_2_ NTs are well-crystallized in the reaction. The WO_2.9_ was also identified after the reaction due to the non-reacted residue of tungsten oxide precursor material.

**Figure 2:**
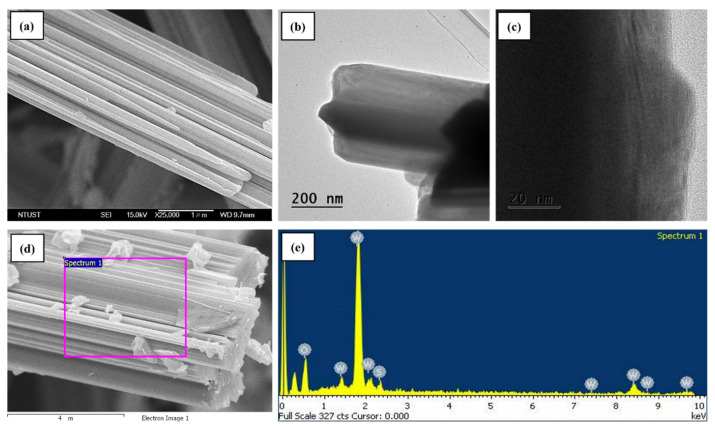
Morphology of WS_2_ NTs: (**a**) SEM image of clustered WS_2_ NTs, (**b**,**c**) TEM images with low and high magnification, and (**d**,**e**) electron image and respective EDS spectrum.

Furthermore, the surface morphology of the as-prepared WS_2_ NTs was observed by FE-SEM, and the images are presented in Figure 2. It can be seen that a large number of NTs formed as clusters with an average dimension of 1.5 to 2 μm in diameter. However, the individual WS_2_ NTs reveal an average diameter of 50 to 90 nm (Figure 2a). Moreover, the TEM images of WS_2_ NTs with high magnification are presented in Figure 2b, showing the successful synthesis of multiwall inorganic NTs. It can be clearly understood that Figure 2c shows the clear boundaries of the inner and outer walls, indicating the successful formation of NTs. The NT structure might provide several active sites and provide enough interaction with magnesium alloy powders during the ball milling process. The small grain size and the nanotube structures facilitated the hydrogenation and dehydrogenation properties of AZ31 Mg alloy powders. Further, the SEM-EDS and mapping analysis were performed for the NT clusters as presented in Figure 2d,e, resulting in the EDS detecting the presence of tungsten (W), sulfur (S), and oxygen (O). The oxygen might be from the minute, unreacted, pristine tungsten oxide (WO_3_) during the nanotube synthesis process.

### 3.2. Morphology Analysis of AZ31-WS_2_ NT/Pd Composite Powders

**Figure 3:**
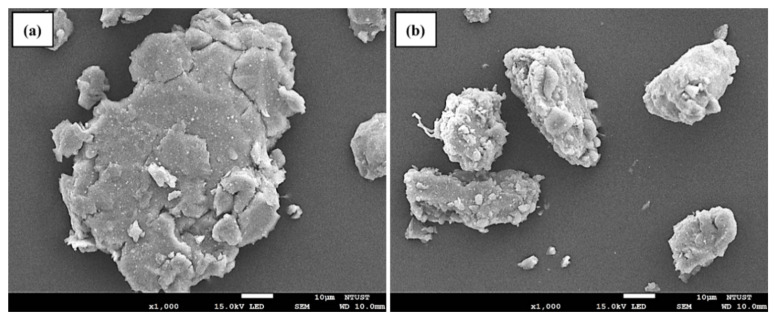
SEM morphologies of AZ31 WS_2_ NT/Pd composite powders: (**a**) as-ball milled and (**b**) after hydrogenation.

**Figure 4:**
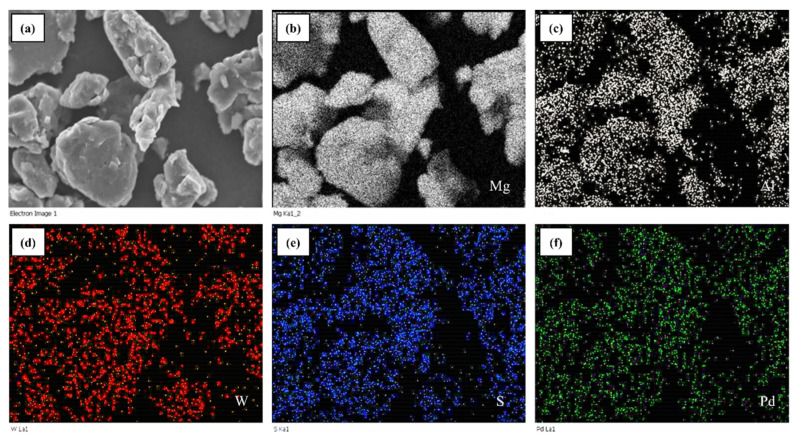
EDS Mapping of (**a**) AZ31 WS_2_ NT/Pd composite, (**b**) Mg, (**c**) Al, (**d**) W, (**e**) S, and (**f**) Pd.

The different quantities of WS_2_ NT/Pd were added to all of the prepared AZ31-WS_2_ NT/Pd composites throughout the 8 h ball milling process. The surface morphology of ball-milled AZ31-8WP before and after hydrogenation was observed by FE-SEM and presented in Figure 3. The powder samples contain an irregular morphology due to the highly uneven force generated during the ball milling process. The particle size was measured for rasped pristine AZ31 samples and is in the range of 80 to 100 μm. When the content of WS_2_ NT/Pd increased from 0 to 8 wt.%, the average particle size of the AZ31-WS_2_ NT composites decreased to 19.7, 16.4, and 15.3 μm, for 0, 4, and 8 wt.%, respectively. This indicates that the particle size of the AZ31 alloy with different quantities of WS_2_ NTs is not noticeably different. Additionally, the elemental dispersion mapping shown in Figure 4, the elements Al, W, S, and Pd in the ball-milled sample are evenly distributed on the magnesium alloy powders with the scale of 10 μm. The uniform distribution serves as one of the significant factors for improving the diffusion of hydrogen along the Mg-alloy interfaces. The ball milling of AZ31 magnesium composite results in a reduction in grain size and creates several distinct defects, which allow the hydrogen atoms to diffuse more quickly [37].

### 3.3. Hydrogen Storage Properties of AZ31-WS_2_ NT/Pd Composites

**Figure 5:**
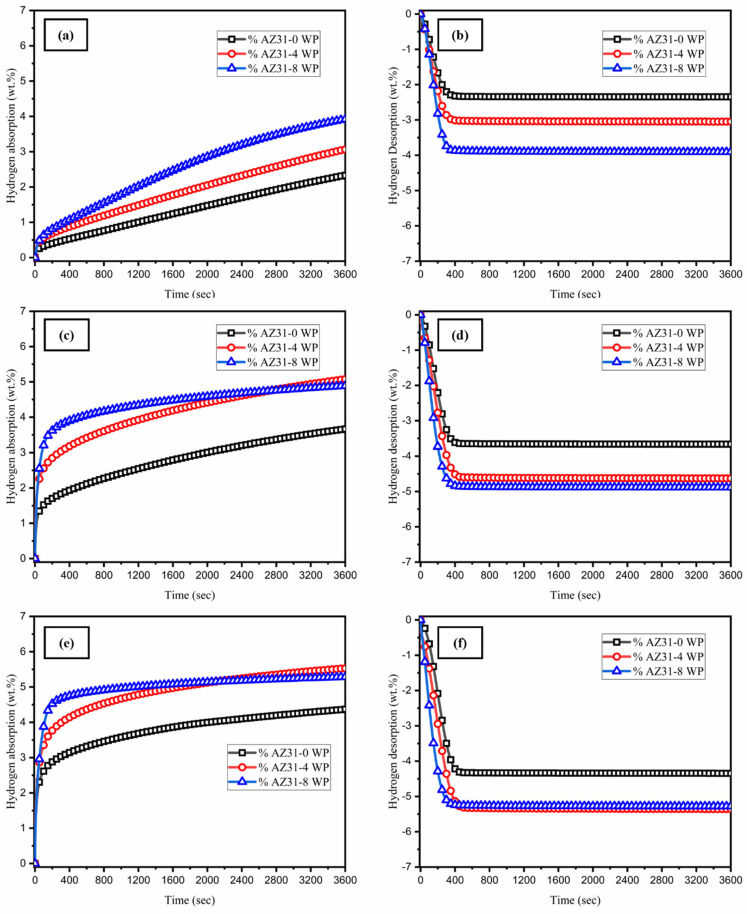
The hydrogen absorption and desorption activation curves of AZ31-WS_2_ NT/Pd composites: (**a**,**b**) first cycle, (**c**,**d**) second cycle, and (**e**,**f**) third cycle.

The activation curves at 375 °C for three different types of AZ31-WS_2_ NT/Pd composite were measured to investigate their hydrogen storage properties. From Figure 5a, the three different types of composites have a linear relationship curve for absorption in the first cycle. The pristine AZ31 magnesium alloy exhibits the slowest rate of hydrogen absorption, while catalyst-added sample powders such as AZ31-4 WP and AZ31-8 WP show a faster absorption. The AZ31-8 WP composite reached its maximum capacity of 3.91 wt.% in 60 min; likely, the AZ31-0 WP and AZ31-4 WP absorbed 2.32 wt.% and 3.06 wt.%, respectively. Obliviously, the addition of WS_2_ NT/Pd catalyst to the AZ31 magnesium increased the rate of nucleation of the hydrogen into the composite powders. Similarly, the rate of hydrogen desorption differs for the three samples, which specifies that the WS_2_-NT/Pd catalyst has an effect on the dehydrogenation kinetics of the AZ31 magnesium alloy. The AZ31 magnesium alloy with a high quantity (8 wt.%) of WS_2_-NT/Pd composite has released the hydrogen faster than other materials in the first activation cycle, which released 3.90 wt.% in 60 min. In the second cycle, the rate of hydrogenation was noticeably different for the three composites: the hydrogen absorption capacity of AZ31-4 WP reached a higher capacity, and the rate of absorption of AZ31-8 WP was faster than that of the other two samples (Figure 5c). During the desorption process, the dehydrogenation rate of the three samples varies from each other, and the entire amount of hydrogen is released in less than 500 s. Notably, the hydrogen absorption and desorption capacities of the second cycle reached a larger quantity than the first cycle. In the third activation cycle, the same scenario as in cycle two is followed: the hydrogen storage capacity of AZ31-4 WP is 0.25 wt.% larger, but the more stable absorption curve observed in AZ31-8 WP reached the maximum capacity of 5.28 wt.% (Figure 5e). However, a decline in hydrogen storage capacity was noted from the third absorption–desorption cycle onward. This reduction is attributed to the high concentration of catalyst inclusion. While the catalyst accelerates the hydrogenation reaction kinetics, it does not directly contribute to hydrogen storage. The excessive catalyst content effectively reduces the available volume of reactive magnesium, thereby limiting the overall hydrogen storage capacity. During the desorption process, no incubation time has been observed for the three materials for the three cycles of activation observed in Figure 5b,d,f. In the activation stage, the hydrogenation curve of the AZ31-8 WP additive sample responds to hydrogen very fast among the three different samples, which implies that increasing the amount of WS_2_ NT/Pd in the alloy samples speeds up the rate of nucleation that is feasible for activation.

**Figure 6:**
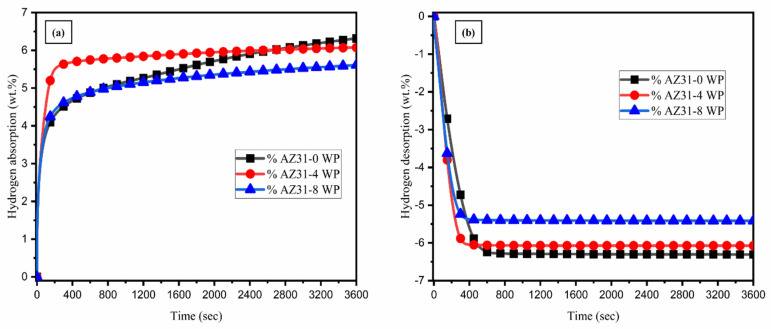
Isothermal hydrogen absorption and desorption kinetics curves of (**a**,**b**) AZ31-WS_2_ NT/Pd composites.

The isothermal hydrogenation and dehydrogenation properties of AZ31 with different WS_2_ NT/Pd content were studied at 375 °C, and the results are shown in Figure 6. The addition of WS_2_NT/Pd accelerates the rate of absorption and desorption. At the temperature of 375 °C, AZ31-0 WP, which has no additives, absorbed 6.31 wt.% hydrogen as the highest, while 6.07 wt.% hydrogen was absorbed for AZ31-4 WP and 5.60 wt.% hydrogen for AZ31-8 WP at the same parameter conditions (Figure 6a). AZ31-0 WP desorbed 6.24 wt.% in 10 min at the fixed temperature level, while AZ31-4 WP desorbed 6.07 wt.% and 5.58 wt.% hydrogen for AZ31-8 WP. The observations illustrated that increasing the quantity of WS_2_NT/Pd content significantly enhanced the hydrogenation and dehydrogenation kinetics of the composites. In addition to that, increasing the quantity of catalyst influences the hydrogen storage capacity significantly. Although the catalyst significantly enhances the hydrogenation reaction kinetics, it does not directly contribute to hydrogen storage. Instead, the excessive catalyst content reduces the volume fraction of reactive magnesium available for hydrogen uptake, thereby limiting the overall storage capacity. The same parameters have been maintained during ball milling of the composite. Therefore, the performance of the different composites was mainly attributed to the varying WS_2_-NT/Pd content. It is a fact that AZ31-4 WP contained fast kinetics with minimal loss of hydrogen storage capacity.

### 3.4. XRD-Phase Analysis of AZ31-WS_2_ NT/Pd Composite

**Figure 7:**
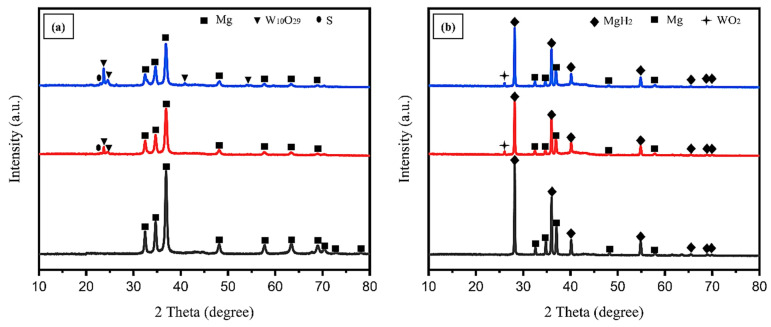
XRD patterns of AZ31-WS_2_ NT/Pd composite: (**a**) as ball milled and (**b**) after hydrogenation process.

The above-mentioned kinetic results show that the addition of WS_2_ NT/Pd composite to the AZ31 magnesium alloy significantly enhanced the rate of hydrogen absorption and desorption. The phase transformations of two different states, such as ball milled and after hydrogenation, were investigated by XRD, and the results are presented in Figure 7. After the ball milling process, the identified Mg phase has sharp peaks for AZ31 magnesium alloy. While adding WS_2_-NT/Pd composite, the Mg peak intensity decreased with the increment of catalyst content. Moreover, the W_10_O_29_ and S (sulfur) phases identified individually show that during the ball milling process, the added materials react with the residual oxygen. The WO_2_ phase was identified after hydrogenation cycles. The presence of numerous oxygen vacancies on WO_2.9_ increases the electronic conductivity properties and also offers more sites for activation [38]. Because it was believed that the presence of WO_2.9_ could significantly facilitate the H_2_ molecules’ dissociation on the surface to enhance the absorption and the presence of transition metal oxide such as WO_2_ plays the role of catalyst during desorption that weaken the bond strength between hydrogen and magnesium enhance the rates of the hydrogen desorption in the storage materials system [39]. Due to the conceivable low quantity, the Pd was not identified in the XRD patterns of the AZ31-WS_2_ NT/Pd composite before and after hydrogenation. Additionally, the residual magnesium presented in the three different compositions demonstrates that the composites have not reached their actual capacity of hydrogenation in 60 min. The comparative research on chalcogenides indicates that the presence of sulfur compounds in magnesium hydrogenation may not have a favorable effect on the hydrogenation capacity’s performance [40]. After the hydrogenation MgH_2_ phase has been formed with a sharp crystalline structure.

The reaction equation presented in Equation (3):

2Mg(s) + H_2_(g) → 2MgH_2_(s) (3)

### 3.5. Activation Energy Calculation

**Figure 8:**
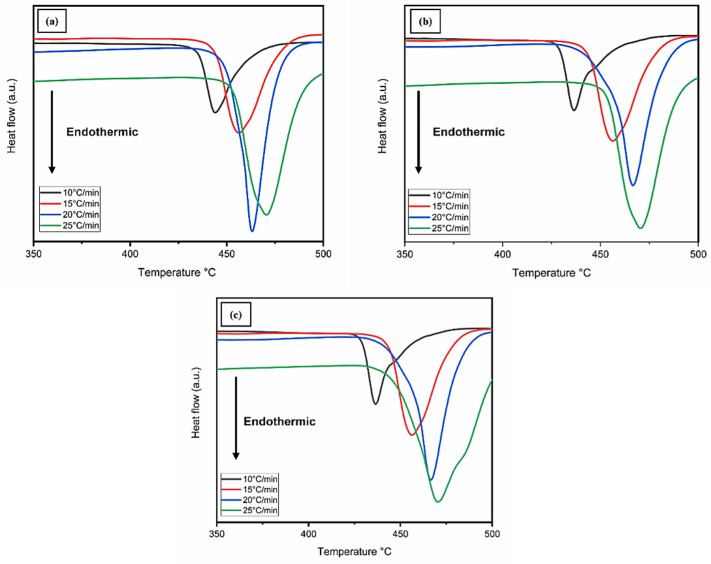
DSC curves of (**a**) AZ31-0 WP, (**b**) AZ31-4 WP, and (**c**) AZ31-8 WP at various heating rates.

**Figure 9:**
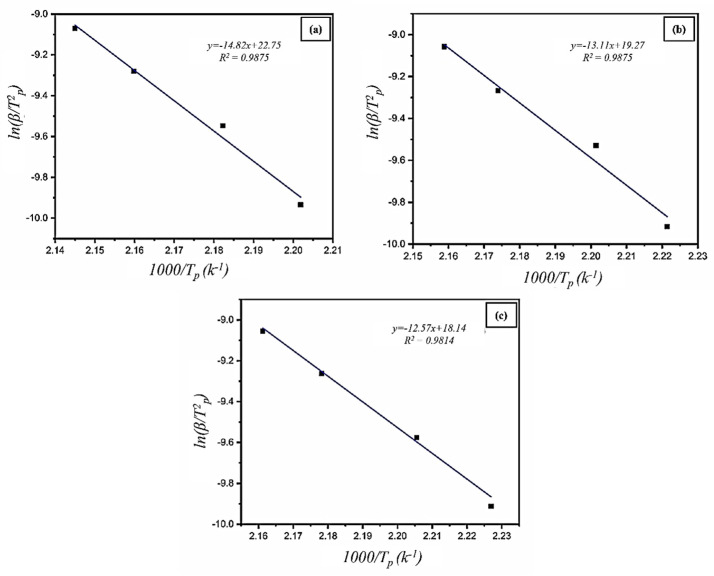
Kissinger plots of (**a**) AZ31-0WP, (**b**) AZ31-4WP, and (**c**) AZ31-8WP obtained from DSC measurement.

Furthermore, the thermal decomposition measurements of three different samples were analyzed. After the hydrogenation process, the powdered samples were subjected to DSC (differential scanning calorimetry) analysis, which was heated up to a maximum temperature of 500 °C. Four different rates of heating (10, 15, 20, and 25 °C/min) were used to investigate the materials, and endothermic peaks are presented in Figure 8. The apparent activation energies (E_a_) of hydrogen desorption for AZ31-WS_2_ NT/Pd composites were calculated by using the Kissinger formula as presented in Equation (4) [41].

ln (β/*T^2^_p_*) = − (*E_a._*/(R*T_p_*)) + C (4)

where β is the heating rate observed from DSC measurements, T_p_ is the peak temperature, C is a temperature-independent constant, and R is an atmospheric standard gas constant. From the Kissinger plot presented in Figure 9, the apparent activation energy values E_a_ for AZ31-WS_2_ NT/Pd composites were evaluated to be 123.25 kJ/mol, 109.06 kJ/mol, and 104.58 kJ/mol for AZ31-0 WP, AZ31-4 WP, and AZ31-8 WP, respectively, which are comparatively much lower than the MgH_2_ storage system 161.9 (kJ/mol) [42]. Table 1 displays some of the magnesium-based alloys and composites. We can see that the activation energy of this work, AZ31-8 WP, is lower than those of some recent results. The decrease in activation energy demonstrated that the inclusion of WS_2_-NT/Pd might lower the energy barrier for hydrogen breakdown in MgH_2_, leading to better hydrogen decomposition kinetics of MgH_2_ in the AZ31-WS_2_ NT/Pd composites.

## 4. Conclusions

In this study, the inorganic WS_2_ NTs were successfully synthesized by using the chemical vapor deposition method. Three distinct compositions of AZ31-WS_2_ NT/Pd have been prepared through ball milling, and the analysis of their microstructures, activation characteristics, hydrogen storage capabilities, and catalytic activities has been investigated. In the course of the activation process, the composite with an increased catalyst concentration (AZ31-8 WP) exhibits more stable and rapid hydrogen absorption and desorption rates. The composite AZ31-4WP demonstrates higher kinetic performance with a maximum storage capacity of 6.07 wt.% during the isothermal de/hydrogenation. The capacity was drastically lowered to 5.60 wt.% by increasing the WS_2_ NT/Pd catalytic content to 8 wt.%. The desorption activation energy was lowered from 123.25 kJ/mol to 104.58 kJ/mol. These improvements in hydrogenation performance are caused by the addition of WS_2_ NT/Pd catalyst to the AZ31-Mg alloy. This could be beneficial in the future development and creation of high-performance hydrogenation materials with the addition of inorganic NTs and transition metal composites on magnesium-based alloys.

## Figures and Tables

**Table 1:** The activation energy of (E_a_) recent Mg-based alloy materials and composites.

Sample	Ea (kJ/mol)	References
MgH_2_ + 5 wt.% Ti-CoO @ C-3	137.76	[43]
Mg_90_ Al_10_	157.7 ± 0.8	[44]
MgH_2_ + CuS_2_	123.7 ± 8	[45]
Mg_88_Y_12_	122.0	[46]
Mg-20 Ce	110.0 ± 3	[47]
Mg_90_Ce_5_Y_5_ + Mn	114.24	[48]
Sm_5_Mg_41_ + 4 wt % NG	112.88	[49]
AZ31-ECAP	128 ± 5	[50]
AZ31-4 WP	109.06	Current work
AZ31-8 WP	104.58	Current work

## Data Availability

Data are contained within the article and Supplementary Materials.

## Supplementary Materials

The following supporting information can be downloaded at: https://www.mdpi.com/article/10.3390/nano15110802/s1, Figure S1: SEM morphologies of AZ31 WS2 NTs/Pd composite powders (a) After hydrogenation, and (b) after hydrogenation Magnified; Figure S2: EDS Mapping of (a) AZ31 WS2 NTs/Pd composite, (b) Mg, (c) Al, (d) W, (e) S, (f) Pd and (g) EDS spectrum.

## References

[1] Shao H., He L., Lin H., Li H. (2018). Progress and Trends in Magnesium-Based Materials for Energy-Storage Research: A Review. Energy Technol..

[2] Kim K.C. (2018). A review on design strategies for metal hydrides with enhanced reaction thermodynamics for hydrogen storage applications. Int. J. Energy Res..

[3] Zhang J., Yan S., Qu H. (2017). Stress/strain effects on thermodynamic properties of magnesium hydride: A brief review. Int. J. Hydrogen Energy.

[4] Zhang X., Liu Y., Hu J., Gao M., Pan H. (2020). Empowering hydrogen storage performance of MgH_2_ by nanoengineering and nano catalysis. Mater. Today Nano.

[5] Zhang J., Yan S., Qu H. (2018). Recent progress in magnesium hydride modified through catalysis and nanoconfinement. Int. J. Hydrogen Energy.

[6] Jain I.P. (2009). Hydrogen the fuel for 21st century. Int. J. Hydrogen Energy.

[7] El-Eskandarany M.S. (2019). Recent developments in the fabrication, characterization and implementation of MgH_2_-based solid-hydrogen materials in the Kuwait Institute for Scientific Research. RSC Adv..

[8] Sadhasivam T., Kim H.-T., Jung S., Roh S.-H., Park J.-H., Jung H.-Y. (2017). Dimensional effects of nanostructured Mg/MgH_2_ for hydrogen storage applications: A review. Renew. Sustain. Energy Rev..

[9] Tian M., Shang C. (2011). Nano-structured MgH_2_ catalyzed by TiC nanoparticles for hydrogen storage. J. Chem. Technol. Biotechnol..

[10] Bhatnagar A., Johnson J.K., Shaz M.A., Srivastava O.N. (2018). TiH_2_ as a Dynamic Additive for Improving the De/Rehydrogenation Properties of MgH_2_: A Combined Experimental and Theoretical Mechanistic Investigation. J. Phys. Chem. C.

[11] Jain I., Lal C., Jain A. (2010). Hydrogen storage in Mg: A most promising material. Int. J. Hydrogen Energy.

[12] Zhang M., Xiao X., Wang X., Chen M., Lu Y., Liu M., Chen L. (2019). Excellent catalysis of TiO_2_ nanosheets with high-surface-energy {001} facets on the hydrogen storage properties of MgH_2_. Nanoscale.

[13] Zhou C., Bowman R.C., Fang Z.Z., Lu J., Xu L., Sun P., Liu H., Wu H., Liu Y. (2019). Amorphous TiCu-Based Additives for Improving Hydrogen Storage Properties of Magnesium Hydride. ACS Appl. Mater. Interfaces.

[14] Chen G., Zhang Y., Chen J., Guo X., Zhu Y., Li L. (2018). Enhancing hydrogen storage performances of MgH_2_ by Ni nano-particles over mesoporous carbon CMK-3. Nanotechnology.

[15] Yuan Z., Sui Y., Zhai T., Yin Y., Luo L., Feng D. (2021). Influence of CeO_2_ nanoparticles on microstructure and hydrogen storage performance of Mg-Ni-Zn alloy. Mater. Charact..

[16] Shtender V., Denys R., Paul-Boncour V., Riabov A., Zavaliy I. (2014). Hydrogenation properties and crystal structure of YMgT_4_ (T = Co, Ni, Cu) compounds. J. Alloys Compd..

[17] Wang J., Zhang W., Cheng Y., Ke D., Han S. (2015). Hydrogenation/dehydrogenation performances of the MgH_2_-WS_2_ composites. J. Wuhan Univ. Technol. Mater. Sci. Ed..

[18] Jia Y., Han S., Zhang W., Zhao X., Sun P., Liu Y., Shi H., Wang J. (2013). Hydrogen absorption and desorption kinetics of MgH_2_ catalyzed by MoS_2_ and MoO_2_. Int. J. Hydrogen Energy.

[19] Xie X., Ma X., Liu P., Shang J., Li X., Liu T. (2017). Formation of Multiple-Phase Catalysts for the Hydrogen Storage of Mg Nanoparticles by Adding Flowerlike NiS. ACS Appl. Mater. Interfaces.

[20] Zhang W., Xu G., Cheng Y., Chen L., Huo Q., Liu S. (2018). Improved hydrogen storage properties of MgH_2_ by the addition of FeS_2_ micro-spheres. Dalton Trans..

[21] Huang S.-J., Rajagopal V., Ali A.N. (2019). Influence of the ECAP and HEBM processes and the addition of Ni catalyst on the hydrogen storage properties of AZ31-x Ni (x = 0, 2, 4) alloy. Int. J. Hydrogen Energy.

[22] Ji L., Zhang L., Yang X., Zhu X., Chen L. (2020). The remarkably improved hydrogen storage performance of MgH_2_ by the synergetic effect of an FeNi/rGO nanocomposite. Dalton Trans..

[23] Ding X., Ding H., Song Y., Xiang C., Li Y., Zhang Q. (2020). Activity-Tuning of Supported Co–Ni Nanocatalysts via Composition and Morphology for Hydrogen Storage in MgH_2_. Front. Chem..

[24] Wang K., Deng Q. (2020). Constructing Core-Shell Co@N-Rich Carbon Additives Toward Enhanced Hydrogen Storage Performance of Magnesium Hydride. Front. Chem..

[25] Shahi R.R., Tiwari A.P., Shaz M., Srivastava O. (2013). Studies on de/rehydrogenation characteristics of nanocrystalline MgH_2_ co-catalyzed with Ti, Fe and Ni. Int. J. Hydrogen Energy.

[26] Tarasov B.P., Mozhzhukhin S.A., Arbuzov A.A., Volodin A.A., Fokina E.E., Fursikov P.V., Lototskyy M.V., Yartys V.A. (2020). Features of the Hydrogenation of Magnesium with a Ni-Graphene Coating. Russ. J. Phys. Chem. A.

[27] Verma S.K., Bhatnagar A., Shukla V., Soni P.K., Pandey A.P., Yadav T.P., Srivastava O.N. (2020). Multiple improvements of hydrogen sorption and their mechanism for MgH_2_ catalyzed through TiH_2_@Gr. Int. J. Hydrogen Energy.

[28] Pukazhselvan D., Sandhya K.S., Ramasamy D., Shaula A., Fagg D.P. (2020). Transformation of Metallic Ti to TiH_2_ Phase in the Ti/MgH_2_ Composite and Its Influence on the Hydrogen Storage Behavior of MgH_2_. Chem. Phys. Chem..

[29] Ma Z., Zou J., Khan D., Zhu W., Hu C., Zeng X., Ding W. (2019). Preparation and hydrogen storage properties of MgH_2_-trimesic acid-TM MOF (TM=Co, Fe) composites. J. Mater. Sci. Technol..

[30] Park K.B., Ko W.-S., Fadonougbo J.O., Na T.-W., Im H.-T., Park J.-Y., Kang J.-W., Kang H.-S., Park C.-S., Park H.-K. (2021). Effect of Fe substitution by Mn and Cr on first hydrogenation kinetics of air-exposed TiFe-based hydrogen storage alloy. Mater. Charact..

[31] Taherkhani A., Mortazavi S., Reyhani A., Tayal A., Caliebe W., Moradi M., Noei H. (2023). Temperature-dependent hydrogen storage mechanism in palladium nanoparticles decorated on multi-walled carbon nanotubes. Int. J. Hydrogen Energy.

[32] Rothschild A., Sloan J., Tenne R. (2000). Growth of WS_2_ nanotubes phases. J. Am. Chem. Soc..

[33] Huang S.-J., Immanuel P.N., Yen Y.-K., Yen C.-L., Tseng C.-E., Lin G.-T., Lin C.-K., Huang Z.-X. (2021). Tungsten disulfide nanotube-modified conductive paper-based Chemi resistive sensor for the application in volatile organic compounds’ detection. Sensors.

[34] Immanuel P.N., Huang S.-J., Danchuk V., Sedova A., Prilusky J., Goldreich A., Shalom H., Musin A., Yadgarov L. (2022). Improving the Stability of Halide Perovskite Solar Cells Using Nanoparticles of Tungsten Disulfide. Nanomaterials.

[35] Immanuel P.N., Huang S.J., Taank P., Goldreich A., Prilusky J., Byregowda A., Carmieli R., Shalom H., Leybovich A., Zak A. (2024). Enhanced photocatalytic activity of Cs_4_PbBr_6_/WS_2_ hybrid nanocomposite. Adv. Energy Sustain. Res..

[36] Huang S.-J., Rajagopal V., Chen Y.L., Chiu Y.H. (2020). Improving the hydrogenation properties of AZ31-Mg alloys with different carbonaceous additives by high energy ball milling (HEBM) and equal channel angular pressing (ECAP). Int. J. Hydrogen Energy.

[37] Bendyna J.K., Dyjak S., Notten P.H. (2015). The influence of ball-milling time on the dehydrogenation properties of the NaAlH_4_-MgH_2_ composite. Int. J. Hydrogen Energy.

[38] Bayeh A.W., Kabtamu D.M., Chang Y.-C., Chen G.-C., Chen H.-Y., Liu T.-R., Wondimu T.H., Wang K.-C., Wang C.-H. (2019). Hydrogen-Treated Defect-Rich W_18_O_49_ Nanowire-Modified Graphite Felt as High-Performance Electrode for Vanadium Redox Flow Battery. ACS Appl. Energy Mater..

[39] Yadav D.K., Chawla K., Pooja, Lal N., Choudhary B., Lal C. (2021). Catalytic effect of TiO_2_on hydrogen storage properties of MgH_2_. Mater. Today Proc..

[40] Wang L., Hu Y., Lin J., Leng H., Sun C., Wu C., Li Q., Pan F. (2022). The hydrogen storage performance and catalytic mechanism of the MgH_2_-MoS_2_ composite. J. Magnes. Alloys.

[41] Gao S., Wang H., Wang X., Liu H., He T., Wang Y., Wu C., Li S., Yan M. (2020). MoSe_2_ hollow nanospheres decorated with FeNi_3_ nanoparticles for enhancing the hydrogen storage properties of MgH_2_. J. Alloys Compd..

[42] Zhang Q., Zang L., Huang Y., Gao P., Jiao L., Yuan H., Wang Y. (2017). Improved hydrogen storage properties of MgH_2_ with Ni-based compounds. Int. J. Hydrogen Energy.

[43] Zhang L., Zhao C., Wu F., Wang Y. (2023). Carbon-wrapped Ti-Co bimetallic oxide nanocages: Novel and efficient catalysts for hydrogen storage in magnesium hydride. J. Alloys Compd..

[44] Li Y., Zhang Y., Shang H., Qi Y., Li P., Zhao D. (2018). Investigation on structure and hydrogen storage performance of as-milled and cast Mg_90_Al_10_ alloys. Int. J. Hydrogen Energy.

[45] Wang P., Tian Z., Wang Z., Xia C., Yang T., Ou X. (2021). Improved hydrogen storage properties of MgH_2_ using transition metal sulfides as catalyst. Int. J. Hydrogen Energy.

[46] Yang T., Yuan Z., Bu W., Jia Z., Qi Y., Zhang Y. (2016). Evolution of the phase structure and hydrogen storage thermodynamics and kinetics of Mg_88_Y_12_ binary alloy. Int. J. Hydrogen Energy.

[47] Xie L., Li J., Zhang T., Song L. (2017). Dehydrogenation steps and factors controlling desorption kinetics of a Mg Ce hydrogen storage alloy. Int. J. Hydrogen Energy.

[48] Wang S., Yong H., Yao J., Ma J., Liu B., Hu J., Zhang Y. (2023). Influence of the phase evolution and hydrogen storage behaviors of Mg-RE alloy by a multi-valence Mo-based catalyst. J. Energy Storage.

[49] Yuan Z., Li C., Li T., Zhai T., Sui Y., Li X., Feng D., Zhang Y. (2023). Improved hydrogen storage performance of Sm-Mg composites by adding nano-graphite. J. Alloys Compd..

[50] Huang S.-J., Rajagopal V., Skripnyuk V., Rabkin E., Fang C. (2023). A comparative study of hydrogen storage properties of AZ31 and AZ91 magnesium alloys processed by different methods. J. Alloys Compd..

