# Supplementary material for: The Hydrogen Storage Properties and Catalytic Mechanism of the AZ31-WS2 Nanotube/Pd Composite
Source: Nanomaterials (Basel). 2025 May 27;15(11):802. doi: 10.3390/nano15110802 (PMC12156327; doi:10.3390/nano15110802)
Supplement: Supplementary file 1 [file nanomaterials-15-00802-s001.zip › nanomaterials-3584735-supplementary.pdf]

Supplementary characterization:

Microscopic observations revealed significant changes in the morphology of the material following ball milling and hydrogenation. The solid composite powder particles exhibited a flake-like morphology, which became increasingly fragmented and rough after hydrogenation. This surface rupture is attributed to internal stresses induced by high-pressure hydrogen exposure, which introduces surface defects.

As shown in **Figure S1**, the particle morphology after hydrogenation displays a notably rougher surface compared to the pre-hydrogenated state. The surface is further characterized by the presence of microcracks and fine particulates. These changes are primarily due to the volumetric expansion associated with magnesium hydride formation, which results in severe plastic deformation and particle fracture. The generation of fresh surface area during this process facilitates improved hydrogen absorption/desorption kinetics.

With continued hydrogenation cycles, these microstructural changes become more pronounced, leading to further particle refinement and microcrack formation, as supported by previous reports. The SEM images illustrate hydrogen diffusion into the alloy and the nucleation and growth of hydride phases. **Figure S2** illustrate the EDS mapping of elemental distributions with the appropriate Pd agloromations in the Composite.

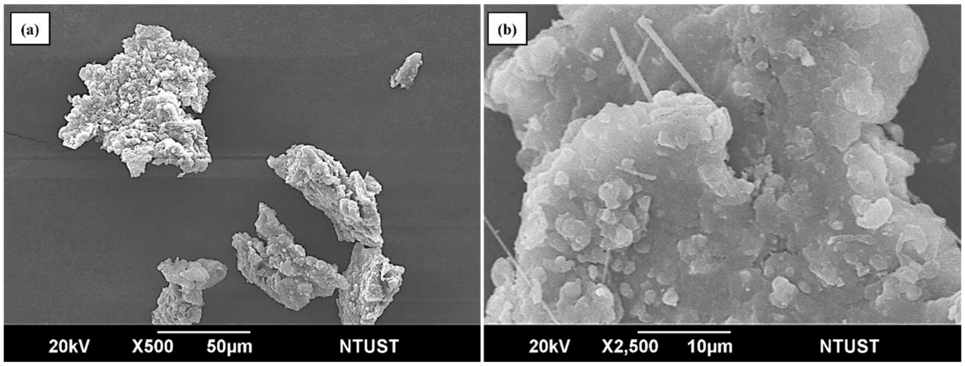

Figure S1. SEM morphologies of AZ31 WS_2 NTs/Pd composite powders (a) After hydrogenation, and (b) after hydrogenation Magnified.

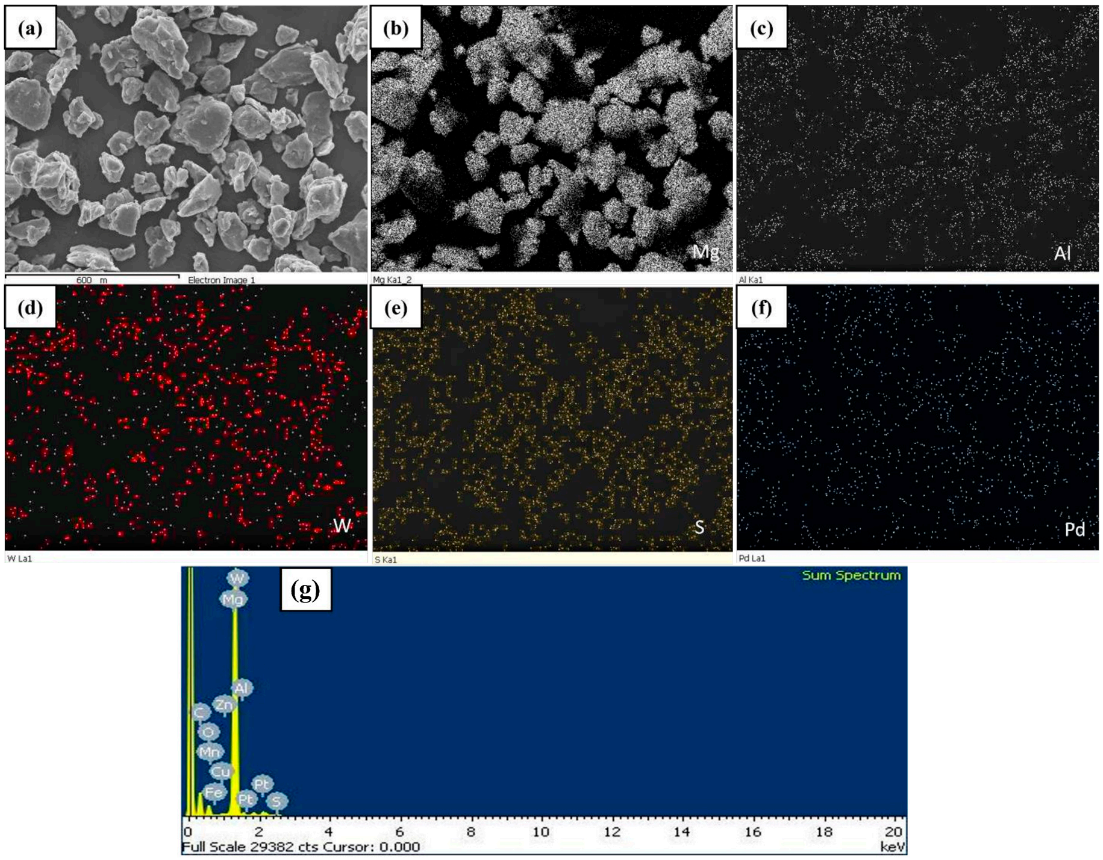

Figure S2. EDS Mapping of (a) AZ31 WS_2 NTs/Pd composite, (b) Mg, (c) Al, (d) W, (e) S, (f) Pd and (g) EDS spectrum.
